# Syk-MyD88 Axis Is a Critical Determinant of Inflammatory-Response in Activated Macrophages

**DOI:** 10.3389/fimmu.2021.767366

**Published:** 2021-12-23

**Authors:** Young-Su Yi, Han Gyung Kim, Ji Hye Kim, Woo Seok Yang, Eunji Kim, Deok Jeong, Jae Gwang Park, Nur Aziz, Suk Kim, Narayanan Parameswaran, Jae Youl Cho

**Affiliations:** ^1^ Department of Integrative Biotechnology, Sungkyunkwan University, Suwon, South Korea; ^2^ Department of Life Sciences, Kyonggi University, Suwon, South Korea; ^3^ Institute of Animal Science, College of Veterinary Medicine, Gyeongsang National University, Jinju, South Korea; ^4^ Department of Physiology and Division of Pathology, Michigan State University, East Lansing, MI, United States

**Keywords:** Syk, MyD88, Src, F-actin, inflammation

## Abstract

**Background:**

Inflammation, a vital immune response to infection and injury, is mediated by macrophage activation. While spleen tyrosine kinase (Syk) and myeloid differentiation primary response 88 (MyD88) are reportedly involved in inflammatory responses in macrophages, their roles and underlying mechanisms are largely unknown.

**Methods:**

Here, the role of the MyD88-Syk axis and the mechanism by which Syk and MyD88 cooperate during macrophage-mediated inflammatory responses are explored using knockout conditions of these proteins and mutation strategy as well as flowcytometric and immunoblotting analyses.

**Results:**

Syk rapidly activates the nuclear factor-kappa B (NF-κB) signaling pathway in lipopolysaccharide (LPS)-stimulated RAW264.7 cells, and the activation of the NF-κB signaling pathway is abolished in Syk^−/−^ RAW264.7 cells. MyD88 activates Syk and Syk-induced activation of NF-κB signaling pathway in LPS-stimulated RAW264.7 cells but Syk-induced inflammatory responses are significantly inhibited in MyD88^−/−^ RAW264.7 cells. MyD88 interacts with Syk through the tyrosine 58 residue (Y58) in the hemi-immunoreceptor tyrosine-based activation motif (ITAM) of MyD88, leading to Syk activation and Syk-induced activation of the NF-κB signaling pathway. Src activates MyD88 by phosphorylation at Y58 *via* the Src kinase domain. In addition, Ras-related C3 botulinum toxin substrate 1 (Rac1) activation and Rac1-induced formation of filamentous actin (F actin) activate Src in LPS-stimulated RAW264.7 cells.

**Conclusions:**

These results suggest that the MyD88-Syk axis is a critical player in macrophage-mediated inflammatory responses, and its function is promoted by an upstream Src kinase activated by Rac1-generated filamentous actin (F-actin).

## Introduction

Inflammation is an innate immune response that protects the body from pathogen infection (1). The inflammatory response initiates by interacting with pattern-recognition receptors expressed on or in inflammatory cells with pathogen-associated molecular patterns (PAMPs) and danger-associated molecular patterns (DAMPs) (1–5), which, in turn, activate inflammatory signaling pathways such as nuclear factor-kappa B (NF-κB), activator protein-1 (AP-1), and interferon regulatory factors (IRFs) (6–10). The NF-κB signaling pathway is one of the most studied pathways among macrophage-mediated inflammatory responses. Activation of the NF-κB signaling pathway is induced by the triggering of fast-signal transduction cascades that stimulate receptor-associated intracellular adaptor molecules, such as myeloid differentiation primary response 88 (MyD88) and TRIF, and numerous intracellular signaling molecules, such as Src, spleen tyrosine kinase (Syk), phosphoinositide 3-kinase (PI3K), Akt, phosphoinositide-dependent kinase 1 (PDK1), *inhibitors *of κB (IκB) kinase α/β (IKKα/β), and IκB in the NF-κB signaling pathway (6, 8, 10). Activation of these molecules in the NF-κB signaling pathway induces inflammatory responses by facilitating the generation of inflammatory mediators, including reactive oxygen species (ROS), nitric oxide (NO) and prostaglandin E_2_; increasing the expression and the activity of inflammatory enzymes and pro-inflammatory cytokines, including caspases, ROS-generating enzymes, inducible NO synthase, cyclooxygenase-2, matrix metalloproteinases, tumor-necrosis factor (TNF)-α, interleukin (IL)-1β, IL-6, interferon (IFN)-β, and interferon-gamma inducible protein 10; and promoting the phagocytic activity of macrophages (6–18).

Syk is a non-receptor type of tyrosine kinase consisting of three main domains: two consecutive Src homology 2 (SH2) domains from the N terminus (SH2-N and SH2-C) and a C-terminal kinase domain (8). Syk is found in many species, including humans, mice, rats, pigs, fruit flies, and hydra, and its molecular structure is highly conserved among species (19–22). Syk is expressed in a wide range of cell types, such as hematopoietic stem cells, immune cells, epithelial cells, vascular endothelial cells, fibroblasts, and neuronal cells (8), and activates numerous intracellular signaling molecules by catalyzing phosphorylation at their tyrosine residues (23), leading to the activation of the NF-κB signaling pathway (8, 24, 25). Syk is a critical player in several biological functions, including innate immunity against pathogens and tissue damage, antibody-dependent cellular cytotoxicity, platelet functions, bone metabolism, vascular development, and cellular adhesion (8, 24).

As a universal adaptor molecule that interacts with the cytoplasmic domains of toll-like receptors (TLRs) and IL-1Rs, MyD88 activates the signal transduction cascades of inflammatory signaling pathways (26, 27). MyD88 is expressed ubiquitously in many types of tissues and consists of three main domains: an N-terminal death domain (DD), an intermediate domain (ID), and a C-terminal toll-interleukin-1 receptor (TIR) domain (27, 28). The TIR domain interacts with other TIR domain-containing proteins, such as TLRs and IL-1Rs, while the DD interacts with IL-1R–associated kinase 1 and 4 (IRAK1 and IRAK4) through homotypic DD interactions (27). The interaction between MyD88 and IRAK1/4 induces IRAK4-mediated IRAK1 phosphorylation, which in turn triggers activation of inflammatory signaling molecules in the NF-κB inflammatory signaling pathway (26–30). A number of studies have demonstrated that MyD88 plays different roles in multiple biological functions, including innate immune responses, carcinogenesis, autophagy, immune activation, learning activity, anxiety, and motor functions (26, 31–37).

Despite a number of previous studies of Syk and MyD88, their biological roles and the underlying molecular mechanisms in macrophages during inflammatory responses remain largely unknown. The functional relationship between Syk and MyD88 in the activation of inflammatory responses in macrophages is also poorly understood. We investigated the roles of Syk and MyD88 and their functional cooperation in macrophage-mediated inflammatory responses. Our findings show that MyD88-Syk axis is a key player in the activation of inflammatory responses through molecular interactions and functional cooperation in macrophages, and that the function of the MyD88-Syk axis is regulated by upstream Src kinase activated by Rac1-induced F-actin formation.

## Materials and Methods

### Materials

Normal C57BL/6 mice (male, 6 weeks, 20-25 g) were purchased from Daehan Bio Link Co., Ltd. (Osong, Korea). MyD88^−/−^ or TRIF^−/−^ mice were obtained from Prof. Narayanan Parameswaran (Department of Physiology, Michigan State University, Michigan, USA). Dietary mouse pellets were purchased from Samyang (Daejeon, Korea). Roswell Park Memorial Institute 1640 (RPMI 1640) cell culture medium, Dulbecco’s modified Eagle’s medium (DMEM), fetal bovine serum (FBS), streptomycin, penicillin, L-glutamine, phosphate-buffered saline (PBS), Lipofectamine 2000, TRIZOL Reagent, MuLV reverse transcriptase, *pfu* DNA polymerase, DH5α-competent cells, polyvinylidene difluoride (PVDF) membrane, enhanced chemiluminescence (ECL) reagent, *pfu* DNA polymerase, and Texas Red-X phalloidin were purchased from Thermo Fisher Scientific (Waltham, MA, USA). Puromycin dihydrochloride, lipopolysaccharide (LPS, *Escherichia coli* 0111:B4), dihydrorhodamine 123 (DHR 123), FITC-E. coli, piceatannol (Pic), Bay 61-3606 (Bay), PP2, bovine serum albumin (BSA), TNF-α, phorbol-12-myristate-13-acetate (PMA), luciferin, sodium dodecyl sulfate (SDS), cytochalasin B (CytoB), and NSC23766 trihydrochloride were purchased from Sigma Chemical Co. (St. Louis, MO, USA). LentiCRISPRv2 plasmid, NF-κB-luciferase reporter gene plasmids, and β-galactosidase plasmids were purchased from Addgene (Cambridge, MA, USA). Antibodies specific for phosphor (p)-IκBα (CST 9246), IκBα (CST 9247), p65 (CST 8242), p50 (CST 12540), p-Syk (CST 2711), Syk (CST 2712), MyD88 (CST 4283), p-Src(Y416) (CST 2101), Src (CST 2109), p-tyrosine (Upstate 05-321), Rac1-GTP (NB 26903), Rac1 (CST 4651), p-p85 (CST 4228), p85 (CST 4292), Flag (CST 8146), Myc (CST 2276), β-actin (CST 4967), and lamin A/C (CST 4777) used for western blot analysis, immunoprecipitation, and immunofluorescence staining were purchased from Abcam (Cambridge, UK), Cell Signaling Technology (Beverly, MA, USA), Santa Cruz Biotechnology, Inc. (Dallas, TX, USA), Upstate Biotechnology (Waltham, MA, USA), and NewEast Biosciences (Malvern, PA, USA). A QIAprep Spin Miniprep Kit was purchased from Qiagen, Inc. (Germantown, MD, USA). Automated DNA sequencing of mutant plasmids was performed at Bionics (Seoul, Korea). sgRNAs of Syk and MyD88 and primers used for quantitative real-time polymerase chain reaction (PCR) amplification were synthesized at Macrogen Inc. (Seoul, Korea). Protein A–coupled Sepharose beads were purchased from GE Healthcare Life Sciences (Marlborough, MA, USA).

### Mice, Husbandry, and Preparation of Peritoneal Macrophages

C57BL/6 mice (male, 6 weeks old, 20 to 25 g) housed in a plastic cage at 22°C in a 12 h light-dark cycle were fed a pelleted mouse diet and tap water *ad libitum*. Animal care and the elements of the study involving mice were conducted according to the guidelines of and in compliance with protocols approved by the Institutional Animal Care and Use Committee at Sungkyunkwan University. Peritoneal exudates were obtained from wild type (WT), MyD88^−/−^ and TRIF^−/−^ C57BL/6 male mice by lavage 4 days after intraperitoneal injection of 1 mL of sterile 4% thioglycolate broth (Difco Laboratories, Detroit, MI, USA), as previously described (38). After washing the exudates with RPMI 1640 medium containing 2% FBS, peritoneal macrophages (1 × 10^6^ cells/mL) were plated in 100 mm tissue culture dishes for 4 h at 37°C in a 5% CO_2_ humidified incubator.

### Cells Culture and Transfection

RAW264.7 and human embryonic kidney 293 (HEK293) cells were purchased from American Type Culture Collection (Rockville, MD, USA). RAW264.7 cells and peritoneal macrophages were culture in RPMI 1640 medium, and HEK293 cells were cultured in DMEM. RPMI 1640 and DMEM were supplemented with 10% heat-inactivated FBS, streptomycin (100 mg/mL), penicillin (100 U/mL), and L-glutamine (2 mM) at 37°C in a 5% CO_2_ humidified incubator. Mycoplasma contamination was regularly tested using the BioMycoX Mycoplasma PCR Detection Kit (CellSafe, Seoul, Korea). RAW264.7 and HEK293 cells were transfected with expression constructs, sgRNA constructs, or siRNA for 48 h using Lipofectamine 2000 according to the manufacturer’s instructions.

### Luciferase Reporter Gene Assay

RAW264.7 or HEK293 cells transfected with NF-κB-luciferase reporter gene constructs and β-galactosidase-expressing constructs were treated or co-transfected with the indicated reagents or constructs using Lipofectamine 2000 according to the manufacturer’s instructions. The cells were lysed by freezing at −70°C and thawing at room temperature three times, and luciferase reporter gene activity was determined by adding luciferin to the cell lysates using a luminometer. All luciferase reporter gene activities were normalized to β-galactosidase activity.

### Preparation of Whole-Cell and Nuclear Lysates

For preparation of whole-cell lysates, RAW264.7, peritoneal macrophages, and HEK293 cells were lysed in ice-cold buffer A (20 mM Tris-HCl, pH 7.4, 2 mM EDTA, 2 mM EGTA, 50 mM glycerol phosphate, 1 mM DTT, 2 µg/mL aprotinin, 2 µg/mL leupeptin, 1 μg/mL pepstatin, 50 μM PMSF, 1 mM benzamide, 2% Triton X-100, 10% glycerol, 0.1 mM sodium vanadate, 1.6 mM pervanadate, and 20 mM NaF) on ice for 30 min, followed by centrifugation at 12,000 rpm at 4°C for 5 min. The supernatant was collected as whole-cell lysates and stored at −20°C until use. To prepare nuclear lysates, RAW264.7 cells were lysed with an ice-cold buffer (10 mM HEPES pH 7.8, 1.5 mM MgCl_2_, 10 mM KCl, 0.5 mM DTT, 0.5% NP-40, 0.1 mM PMSF, 2 μg/mL leupeptin, and 2 μg/mL aprotinin) on ice for 10 min, followed by centrifugation at 3,000 rpm at 4°C for 10 min, after which the supernatant was discarded. The pellet was resuspended in ice-cold buffer B (5 mM HEPES, 1.5 mM MgCl_2_, 0.2 mM EDTA, 0.5 mM DTT, 26% glycerol [v/v], 300 mM NaCl, pH 7.9), followed by sonication for 10 s on ice, and the lysates were centrifuged at 20,000 g at 4°C for 30 min. The supernatant was collected as nuclear lysates and stored at −20°C until use.

### Western Blot Analysis and Immunoprecipitation

For western blot analysis, whole-cell and nuclear lysates were subjected to sodium dodecyl sulfate polyacrylamide gel electrophoresis and transferred on PVDF membranes. The membranes were blocked by a buffer (5% BSA in Tris-buffered saline) at room temperature for 1 h and incubated with primary antibodies specific for each target at room temperature for 1 h. After washing the membranes with a buffer of Tris-base, NaCl, and 0.1% Tween 20 at pH 7.6 three times for 10 min each, the membranes were incubated with horseradish peroxidase–linked secondary antibodies at room temperature for 1 h. After washing three times, the target proteins were visualized by the ECL reagent.

For immunoprecipitation, whole-cell lysates (500 μg exogenous and 1 mg endogenous protein) were pre-cleared using 10 μL of protein A–coupled Sepharose beads (50% v/v) at 4 °C for 1 h, and the pre-cleared whole-cell lysates were incubated with primary antibodies specific for each target at 4°C overnight with gentle rotation. The immunocomplexes were then incubated with protein A–coupled Sepharose beads (50% v/v) at 4°C for 4 h with gentle rotation, and the supernatant was removed from the beads by centrifugation. The beads were washed with ice-cold buffer A five times and boiled for 5 min in protein sample buffer (50 mM Tris-HCl pH 6.8, 2% SDS, 10% glycerol, 1% β-mercaptoethanol, 12.5 mM EDTA, 0.02% bromophenol blue). Immunoprecipitates were analyzed by western blots to detect target proteins.

### Generation of Syk^−/−^ and MyD88^−/−^ RAW264.7 Cells by CRISPR Cas9

Small-guide RNA (sgRNA) sense and anti-sense oligos of Syk and MyD88 were synthesized, and their sgRNA constructs were generated by inserting each sgRNA hybrid into a lentiCRISPRv2 vector, as described previously (39, 40). RAW264.7 cells were transfected with sgRNA constructs using Lipofectamine 2000 according to the manufacturer’s instructions, and transfected RAW264.7 cells were selected using puromycin (1.0 mg/mL) in RPMI 1640 medium containing 10% heat-inactivated FBS until all non-transfected RAW264.7 cells were dead. Syk and MyD88 sgRNA sequences are listed in **Table 1**.

**Table 1:** The sequences of sgRNA used in this study.

Target		Sequence (5′ to 3′)
Syk	Sense	TCGCAATTACTACTACGACG
	Anti-sense	CGTCGTAGTAGTAATTGCGA
MyD88	Sense	TTGGTCGCGCTTAACGTGGG
	Anti-sense	CCCACGTTAAGCGCGACCAA

### Quantitative Real-Time Polymerase Chain Reactions

Total RNA was extracted using TRIZOL reagent according to the manufacturer’s instructions and immediately stored at −70°C until use. A 1 μg sample of the total RNA was used for synthesizing cDNA using MuLV reverse transcriptase, and the cDNA was used for quantitative real-time PCR amplification using SYBR Premix ex Taq according to the manufacturer’s instructions. Primer sequences used for quantitative real-time PCR are listed in **Table 2**.

**Table 2:** Primer sequences used for quantitative real-time PCR in this study.

Target	Primer	Sequence (5 ′ to 3 ′)
iNOS	For	GGAGCCTTTAGACCTCAACAGA
	Rev	TGAACGAGGAGGGTGGTG
NOX1	For	ATCTTCGCGAAACCCGTGAT
	Rev	AGAATCACAGCGAGATGGCTT
AOX1	For	CAACCTTCCATCCAACACTG
	Rev	CCACATTTGATTGCCACTTC
XDH	For	GCGATTGCTACACTTCCAGA
	Rev	AGGGTTGAGGTCAGAGATGG
GAPDH	For	CAATGAATACGGCTACAGCAAC
	Rev	AGGGAGATGCTCAGTGTTGG

### Generation of Syk, MyD88, and Src Mutant Constructs

Syk domain-deletion mutants 1) N-terminal Src homology-2 domain (SH2) deletion mutant (Myc-Syk[ΔSH2-N]), 2) C-terminal SH2 deletion mutant (Myc-Syk[ΔSH2-C]), and 3) kinase domain (KD) deletion mutant (Myc-Syk[ΔKD]); MyD88 domain-deletion mutants 1) death domain (DD) deletion mutant (Flag-MyD88[ΔDD]), 2) intermediate domain (ID) deletion mutant (Flag-MyD88[ΔID]), 3) toll-interleukin-1 receptor domain (TIR) deletion mutant [Flag-MyD88(ΔTIR)], and 4) hemi-immunoreceptor tyrosine-based activation motif (ITAM) deletion mutant (Flag-MyD88 [ΔITAM]), a Src KD domain-deletion mutant (Src[ΔKD]), and a MyD88 tyrosine 58 residue point mutant (Flag-MyD88[Y58F]) were generated by site-directed mutagenesis. Mutant plasmids were generated by PCR amplification using *pfu* DNA polymerase with mutant primers, in PCR conditions of pre-denaturation (95°C, 30 s) followed by 18 cycles of denaturation (95°C, 30 s), annealing (55°C, 1 min), and extension (68°C, 1 min/kb). The PCR-amplified mutant plasmids were transformed into DH5α-competent cells, and the transformed DH5α-competent cells were grown on lysogeny broth agar plates containing ampicillin (100 μg/mL) at 37°C overnight. Mutant plasmids were prepared from the DH5α-competent cells using the QIAprep Spin Miniprep Kit according to the manufacturer’s instructions, and mutagenesis was confirmed by automated DNA sequencing.

### Actin Polymerization Assay

RAW264.7 cells were treated with LPS (1 μg/mL) and Texas Red-X phalloidin (200 units/mL) in the presence or absence of NSC23766 (100 μM) for the indicated times. After washing the cells three times with PBS, the cells were resuspended in a FACS buffer (2% BSA, 0.1% sodium azide in PBS), and the fluorescence of the cells was determined using a flow cytometer.

### Statistical Analysis

The data presented in this study are expressed as the mean ± standard deviation of at least three independent experiments. For statistical comparisons, all results were analyzed by either analysis of variance or the Mann–Whitney test, and a *P* value of less than 0.05 was considered statistically significant (*P < 0.05, **P < 0.01). All statistical analyses were conducted using SPSS software (SPSS Inc., Chicago, IL, USA).

## Results

### Syk Was a Prime Activator of NF-κB Signaling Pathway in Macrophages

**Figure 1:**
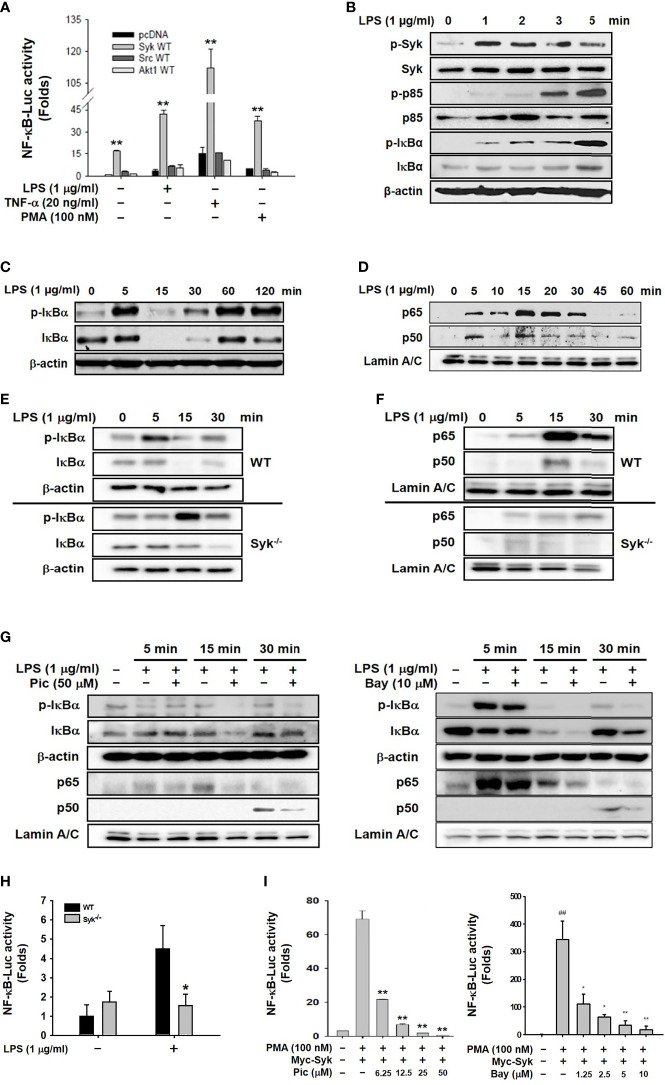
Syk was a prime activator of the NF-κB signaling pathway in macrophages. **(A)** RAW264.7 cells co-transfected with the NF-κB-Luciferase reporter gene plasmid and either empty (pcDNA), Syk, Src, or Akt plasmids were treated with either LPS (1 μg/mL), TNF-α (20 ng/mL), or PMA (100 nM) for 24 h. Luciferase activity was measured by a luminometer and normalized to β-galactosidase activity. **(B, C)** Syk, p-Syk, p85, p-p85, IκBα, and p-IκBα in whole-cell lysates of RAW264.7 cells treated with LPS (1 μg/mL) for the indicated time were detected by western blot analyses. **(D)** p65 and p50 in the nuclear lysates of RAW264.7 cells treated with LPS (1 μg/mL) for the indicated time were detected by western blot analysis. **(E)** IκBα and p-IκBα in the whole-cell lysates of WT and Syk^−/−^ RAW264.7 cells treated with LPS (1 μg/mL) for the indicated time were detected by Western blot analysis. **(F)** p65 and p50 in the nuclear lysates of WT and Syk^−/−^ RAW264.7 cells treated with LPS (1 μg/mL) for the indicated time were detected by western blot analysis. **(G)** IκBα and p-IκBα in the whole-cell lysates of RAW264.7 cells and p65 and p50 in the nuclear lysates of RAW264.7 cells treated with LPS (1 μg/mL) for the indicated time in the absence or presence of Pic (50 μM) and Bay (10 μM) were determined by western blot analysis. **(H)** WT and Syk^−/−^ RAW264.7 cells transfected with the NF-κB-luciferase reporter gene plasmids for 24 h were treated with LPS (1 μg/mL) for 24 h, after which the luciferase activity of these cells was measured by a luminometer and normalized to β-galactosidase activity. **(I)** HEK293 cells co-transfected with the NF-κB-Luciferase reporter gene plasmids and Myc-Syk plasmids for 24 h were treated with PMA (100 nM), Pic (0–50 μM), and Bay (0-10 μM), and the luciferase activity was measured by a luminometer and normalized to β-galactosidase activity. *P < 0.05, **P < 0.01 compared with controls.

We first attempted to identify a prime activator of the NF-κB signaling pathway in macrophages during inflammatory responses. Because Syk, Src, and Akt reportedly induce inflammatory responses in macrophages by activating the NF-κB signaling pathway (6, 8, 10), NF-κB-mediated luciferase reporter gene activity was examined in RAW264.7 cells transfected with constructs expressing Syk, Src and Akt. Syk most strongly induced the NF-κB-mediated luciferase reporter gene activity compared with Src and Akt1 in the RAW264.7 cells under stimulation from NF-κB stimulators, LPS, TNF-α, and PMA (**Figure 1A**). We next investigated the role of Syk in the activation of the NF-κB signaling pathway in macrophages during inflammatory responses. Syk was immediately phosphorylated at 1 min, and the phosphorylation of p85 and IκBα followed at 3 min and 5 min, respectively, in LPS-stimulated RAW264.7 cells (**Figure 1B**). IκBα markedly phosphorylated at 5 min and then degraded from 15 min to 30 min in LPS-stimulated RAW264.7 cells (**Figure 1C**). In accordance with IκBα degradation, nuclear translocation of NF-κB subunits p65 and p50 was initiated at 5 min, peaked at 15 min and lasted for 30 min in LPS-stimulated RAW264.7 cells (**Figure 1D**). IκBα phosphorylation was notably delayed (**Figure 1E**), and the nuclear translocation of p65 and p50 was also markedly delayed in the in the Syk^−/−^ RAW264.7 cells (**Figure 1F**). These results confirmed that the selective Syk inhibitor Pic (50 μM) and Bay (10 μM) inhibited IκBα phosphorylation and nuclear translocation of p65 and p50 in the LPS-stimulated RAW264.7 cells (**Figure 1G**). Also, NF-κB-mediated luciferase reporter gene activity induced by LPS was significantly suppressed in the Syk^−/−^ RAW264.7 cells (**Figure 1H**) and the Pic and Bay-treated HEK293 cells in a dose-dependent manner (**Figure 1I**). These results suggest that Syk is a prime activator of the NF-κB signaling pathway in macrophage-mediated inflammatory responses.

### MyD88 Activated Syk-Induced NF-κB Signaling Pathway in Macrophages

**Figure 2:**
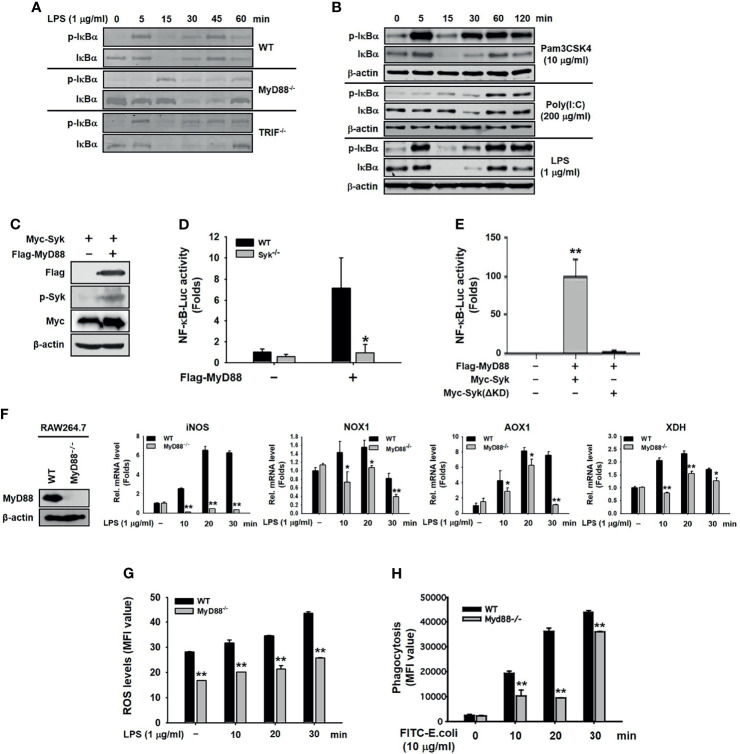
MyD88 activated the Syk-induced NF-κB signaling pathway in macrophages. **(A)** IκBα,and p-IκBα were detected by western blot analysis in the peritoneal macrophages extracted from WT, MyD88^−^/^−^, and TRIF^−/−^ mice under LPS (1 μg/mL) treatment. **(B)** IκBα and p-IκBα in whole-cell lysates of RAW264.7 cells treated with Pam3CSK4 (10 μg/mL), Poly I:C (200 μg/mL), or LPS (1 μg/mL) for the indicated time were detected by western blot analysis. **(C)** p-Syk, Flag, and Myc were detected by western blot analysis in whole-cell lysates of HEK293 cells co-transfected with Myc-Syk and either empty (pcDNA) or Flag-MyD88 plasmid for 48 h. **(D)** WT and Syk^−/−^ RAW264.7 cells were co-transfected with the NF-κB-Luciferase reporter gene plasmid and MyD88 plasmid for 48 h. The luciferase activity of these cells was measured by a luminometer and normalized to β-galactosidase activity. **(E)** HEK293 cells co-transfected with the NF-κB-Luciferase reporter gene plasmid, either empty (pcDNA) or Flag-MyD88 plasmids, and empty (pcDNA) or Myc-Syk ΔKD plasmids for 48 h, and the luciferase activity was measured by a luminometer and normalized to β-galactosidase activity. **(F)** MyD88^−/−^ RAW264.7 cells were generated by CRISPR Cas9, and expression of MyD88 was detected by western blot analysis. WT RAW264.7 and MyD88^−/−^ RAW264.7 cells were treated with LPS (1 μg/mL) for the indicated time, and mRNA expression of the target genes was determined by quantitative real time PCR. **(G)** WT RAW264.7 and MyD88^−/−^ RAW264.7 cells treated with LPS (1 μg/mL) for the indicated time were incubated with DHR 123 (20 μM) for 20 min, and the ROS level was determined by measuring fluorescence. **(H)** WT RAW264.7 and MyD88^−/−^ RAW264.7 cells were incubated with fluorescein isothiocyanate–E. coli (10 μg/mL) for the indicated time, and fluorescence was determined by a fluorescence plate reader. *P < 0.05, **P < 0.01 compared with controls.

We next investigated the role of MyD88 in Syk-activated inflammatory responses in macrophages. MyD88 and TRIF are adaptors that bind to the cytoplasmic domains of TLR4 and induce the activation of TLR4-mediated NF-κB signaling pathways during the innate immune response (8, 41). LPS-induced phosphorylation of IκBα was markedly delayed only in MyD88^−/−^ peritoneal macrophages, and not in TRIF^−/−^ cells (**Figure 2A**). Moreover, the phosphorylation of IκBα was immediately induced in LPS-stimulated and Pam3CSK4-stimulated RAW264.7 cells, but delayed in poly(I:C)-stimulated RAW264.7 cells (**Figure 2B**). The direct effect of MyD88 on Syk-activated inflammatory responses in macrophages was examined further. Co-transfection of HEK293 cells with Myc-Syk and Flag-MyD88 revealed that MyD88 induced Syk phosphorylation (**Figure 2C**). In addition, MyD88-induced NF-κB luciferase reporter gene activity was significantly suppressed in Syk^−/−^ RAW264.7 cells (**Figure 2D**) and in HEK293 cells transfected with Syk kinase-domain mutant constructs (Myc-Syk[ΔKD]) (**Figure 2E**). Syk induces the generation of ROS by increasing the expression of ROS-generating enzymes in macrophages, leading to the induction of phagocytic activity of macrophages (unpublished data). Because MyD88 activated Syk-induced inflammatory responses in macrophages (**Figures 2A–E**), we examined the effects of MyD88 on the expression of ROS-generating enzymes, ROS generation, and the phagocytic activity in macrophages were examined. As expected, the expression of ROS-generating enzymes (**Figure 2F**), ROS generation (**Figure 2G**), and the phagocytic activity of macrophages (**Figure 2H**) were significantly inhibited in MyD88^−/−^ RAW264.7 cells. These results suggest that MyD88 is an upstream activator of Syk in macrophages, leading to the NF-κB pathway activation, ROS generation by upregulating the expression of ROS-generating enzymes, and phagocytosis.

### MyD88 Interacted With Syk in Macrophages

**Figure 3:**
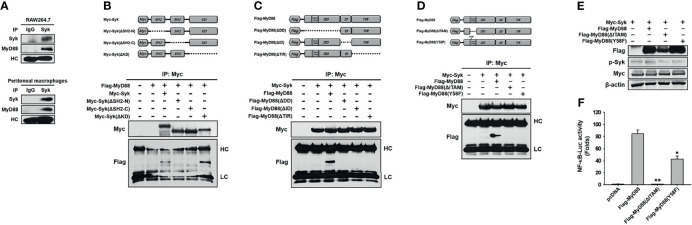
MyD88 interacted with Syk in macrophages. **(A)** Whole-cell lysates of RAW264.7 cells and peritoneal macrophages were immunoprecipitated with either non-specific control immunoglobulin G or Syk antibodies, followed by detection of Syk and MyD88 by western blot analysis. **(B)** Schematic illustration of WT Myc-Syk and Syk deletion mutants, Myc-Syk(ΔSH2-N), Myc-Syk(ΔSH2-C), and Myc-Syk(ΔKD). Whole-cell lysates of HEK293 cells co-transfected with Flag-MyD88 and either Myc-Syk or Syk-deletion mutant plasmids for 48 h were immunoprecipitated with Myc antibodies, followed by detection of Myc and Flag by western blot analysis. **(C)** Schematic illustration of WT Flag-MyD88 and MyD88 deletion mutants, Flag-MyD88(ΔDD), Flag-MyD88(ΔID), and Flag-MyD88(ΔTIR). Whole-cell lysates of HEK293 cells co-transfected with Myc-Syk and either Flag-MyD88 or MyD88 deletion mutant plasmids for 48 h were immunoprecipitated with Myc antibodies, followed by detection of Myc and Flag by western blot analysis. **(D)** Schematic illustration of a WT Flag-MyD88, a MyD88 deletion mutant, Flag-MyD88(ΔITAM), and a point mutant. Whole-cell lysates of HEK293 cells co-transfected with Myc-Syk and either Flag-MyD88, Flag-MyD88(ΔITAM) or Flag-MyD88(Y58F) plasmids for 48 h were immunoprecipitated with Myc antibodies, followed by detection of Myc and Flag by western blot analysis. **(E)** p-Syk, Flag, and Myc in the whole-cell lysates of HEK293 cells co-transfected with Myc-Syk and either Flag-MyD88, Flag-MyD88(ΔITAM), or Flag-MyD88(Y58F) plasmids for 48 h were detected by Western blot analysis. **(F)** HEK293 cells co-transfected with the NF-κB-luciferase reporter gene plasmid and either empty (pcDNA), Flag-MyD88, Flag-MyD88(ΔITAM) or Flag-MyD88(Y58F) plasmids for 48 h, and the luciferase activity was measured by a luminometer and normalized to β-galactosidase activity. *P < 0.05, **P < 0.01 compared with controls.

We then investigated the molecular mechanism by which MyD88 activates Syk in macrophages during inflammatory responses. First, molecular interaction between Syk and MyD88 was examined, and MyD88 interacted with Syk in RAW264.7 cells and peritoneal macrophages (**Figure 3A**). For interaction-site mapping, domain-deletion mutants of Syk (**Figure 3B**) and MyD88 (**Figure 3C**) were generated, and their interaction was examined by co-transfecting HEK293 cells with the mutant plasmids. MyD88 interacted with WT Syk and Myc-Syk(ΔKD), but not with Myc-Syk(ΔSH2-N) and Myc-Syk(ΔSH2-C) (**Figure 3B**). Syk interacted with WT MyD88, but not with any of the three MyD88-deletion mutants, specifically Flag-MyD88(ΔDD), Flag-MyD88(ΔID), and Flag-MyD88(ΔTIR) (**Figure 3C**). Because MyD88 DD contains a hemi-ITAM that itself contains a tyrosine 58 residue, the MyD88 hemi-ITAM deletion mutant (Flag-MyD88[ΔITAM]) and the MyD88 tyrosine 58 residue mutant (Flag-MyD88[Y58F]) were generated (**Figure 3D**), and we examined the interaction between Syk and these MyD88 mutants further. Syk interacted only with WT MyD88, but did not interact with either Flag-MyD88(ΔITAM) or Flag-MyD88(Y58F) (**Figure 3D**). The phosphorylation of Syk and the Syk-activated NF-κB-luciferase reporter gene activity induced by WT MyD88 were suppressed significantly by Flag-MyD88(ΔITAM) and Flag-MyD88(Y58F) (**Figures 3E, F**). These results suggest that molecular interaction is required for MyD88-induced Syk activation, and Y58 in the hemi-ITAM of MyD88 is critical to the interaction with Syk and the consequent Syk activation in macrophages during inflammatory responses.

### Src Activated MyD88 by Phosphorylating Tyrosine 58 Residues in Macrophages

**Figure 4:**
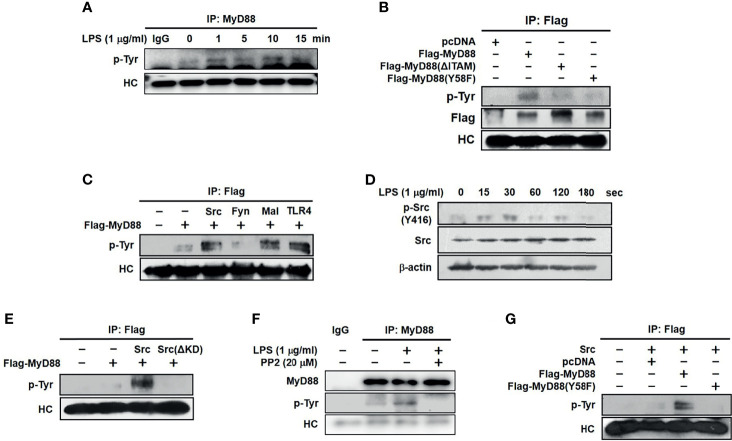
Src activated MyD88 by phosphorylating 58 tyrosine residue in macrophages. **(A)** p-Tyr MyD88 in whole-cell lysates of RAW264.7 cells treated with LPS (1 μg/mL) for the indicated time was detected by immunoprecipitation and western blot analysis. **(B)** p-Tyr MyD88 and Flag in whole-cell lysates of RAW264.7 cells transfected with empty (pcDNA), Flag-MyD88, Flag-MyD88(ΔITAM) or Flag-MyD88(Y58F) plasmids for 48 h were detected by immunoprecipitation and western blot analysis. **(C)** p-Tyr MyD88 in whole-cell lysates of RAW264.7 cells co-transfected with Flag-MyD88 and empty (pcDNA), Src, Fyn, Mal, or TLR4 for 48 h was detected by immunoprecipitation and western blot analysis. **(D)** Src and p-Src in whole-cell lysates of RAW264.7 cells treated with LPS (1 μg/mL) for the indicated time were detected by western blot analysis **(E)** p-Tyr MyD88 in whole-cell lysates of RAW264.7 cells co-transfected with Flag-MyD88 and empty (pcDNA), Src, or Src(ΔKD) was detected by immunoprecipitation and western blot analysis. **(F)** p-Tyr MyD88 in whole-cell lysates of RAW264.7 cells treated with LPS (1 μg/mL) for the 1 min with absence or presence of PP2 (20 μM) was detected by immunoprecipitation and western blot analysis **(G)** p-Tyr MyD88 in whole-cell lysates of RAW264.7 cells co-transfected with Src and either empty (pcDNA), Flag-MyD88, or Flag-MyD88(Y58F) plasmids for 48 h were detected by immunoprecipitation and western blot analysis.

To examine whether MyD88 is activated by phosphorylation in macrophages, MyD88 phosphorylation was evaluated in LPS-stimulated RAW264.7 cells. MyD88 rapidly phosphorylated within 1 min, with phosphorylation decreasing gradually thereafter (**Figure 4A**). As tyrosine is one of the amino acids that are phosphorylated, we then examined whether MyD88 is phosphorylated at the Y58 residue in the hemi-ITAM. RAW264.7 cells were transfected with WT Flag-MyD88 and two MyD88 mutants, Flag-MyD88(ΔITAM) and Flag-MyD88(Y58F). We found that WT MyD88, but neither Flag-MyD88(ΔITAM) nor Flag-MyD88(Y58F) mutants, was phosphorylated (**Figure 4B**). To identify the molecule that phosphorylates MyD88 in macrophages, MyD88 phosphorylation was examined in RAW264.7 cells transfected with TLR4, Mal, and the Src family kinases Src and Fyn. TLR4, Mal, and Src (but not Fyn), phosphorylated MyD88 (**Figure 4C**). Src phosphorylated more rapidly than MyD88 from 15 to 30 s, and its phosphorylation was notably inhibited beyond 30 s in LPS-stimulated RAW264.7 cells (**Figure 4D**). We therefore investigation Src-mediated MyD88 phosphorylation in RAW264.7 cells. MyD88 was phosphorylated by WT Src, but not by Src(ΔKD) (**Figure 4E**). And phosphorylation of MyD88 was inhibited by a selective inhibitor of Src, PP2 (**Figure 4F**). Because MyD88 was phosphorylated at the tyrosine 58 residue in hemi-ITAM by LPS (**Figure 4B**), whether Src phosphorylates MyD88 at the tyrosine 58 residue was examined further, and MyD88 was phosphorylated by Src at Y58 (**Figure 4G**). These results suggest that Src activates MyD88 by phosphorylating the Y58 residue of MyD88 in macrophages during inflammatory responses.

### Rac1 Activated Src by Promoting Actin-Filament Production in Macrophages

**Figure 5:**
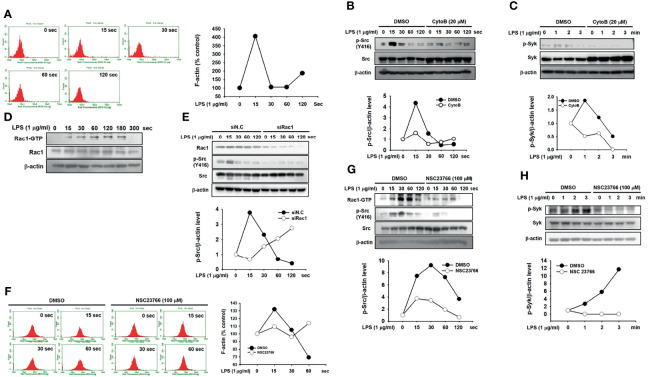
Rac1 activated Src by promoting actin filament production in macrophages. **(A)** p-Tyr MyD88 in whole-cell lysates of RAW264.7 cells treated with LPS (1 μg/mL) for the indicated time was detected by immunoprecipitation and western blot analysis. **(B)** Src and p-Src in whole-cell lysates of RAW264.7 cells incubated with vehicle (DMSO) or CytoB (20 μM), followed by the treatment with LPS (1 μg/mL) for the indicated time were detected by western blot analysis, and the p-Src/β-actin level was plotted. **(C)** p-Syk in whole-cell lysates of RAW264.7 cells incubated with the vehicle (DMSO) or CytoB (20 μM), followed by treatment of LPS (1 μg/mL) for the indicated time were detected by western blot analysis, and the p-Syk/β-actin level was plotted. **(D)** Rac1-GTP and Rac1 in whole-cell lysates of RAW264.7 cells treated with LPS (1 μg/mL) for the indicated time were detected by western blot analysis. **(E)** Rac1, Src, and p-Src in whole-cell lysates of RAW264.7 cells transfected with siN.C or siRac1 for 24 h, followed by the treatment of LPS (1 μg/mL) for the indicated time, were detected by western blot analysis, and the p-Src/Src level was plotted. **(F)** RAW264.7 cells incubated with DMSO or NSC23766 (100 μM) were treated with LPS (1 μg/mL) for the indicated time in the presence of Texas Red-X phalloidin (200 units/mL), and fluorescence was determined with a flow cytometer and plotted. **(G)** Rac1-GTP, Src, and p-Src in the whole-cell lysates of RAW264.7 cells incubated with DMSO or NSC23766 (100 μM), followed by the treatment of LPS (1 μg/mL) for the indicated time were detected by western blot analysis, and the p-Src/Src level was plotted. **(H)** Rac1-GTP, Syk, and p-Syk in whole-cell lysates of RAW264.7 cells incubated with DMSO or NSC23766 (100 μM), followed by treatment of LPS (1 μg/mL) for the indicated time were detected by western blot analysis, and the p-Syk/Syk level was plotted.

Finally, we investigated the molecular mechanism by which Src is activated in macrophages during inflammatory responses. The formation of F actin by actin polymerization increased at 15 s and dramatically decreased after 30 s in LPS-stimulated RAW264.7 cells (**Figure 5A**). To examine whether actin polymerization activates Src in macrophages, RAW264.7 cells were treated with CytoB, an actin polymerization inhibitor (42). Src was highly phosphorylated from 15 to 30 s, while Src phosphorylation was markedly inhibited by CytoB in LPS-stimulated RAW264.7 cells (**Figure 5B**). As with Src, Syk phosphorylation was also inhibited by CytoB in LPS-stimulated RAW264.7 cells (**Figure 5C**). The role of Rac1, a Rho-GTPase on Src activation was next investigated in macrophages. LPS induced Rac1 activation by increasing Rac1-GTP levels in RAW264.7 cells (**Figure 5D**), and Rac1 inhibition by Rac1-specific siRNA (siRac1) notably suppressed Src phosphorylation in LPS-stimulated RAW264.7 cells (**Figure 5E**). Inhibition of Rac1 activation by its selective inhibitor, NSC23766, suppressed F-actin formation (**Figure 5F**) and phosphorylation of Src (**Figure 5G**) and Syk (**Figure 5H**) in LPS-stimulated RAW264.7 cells. These results suggest that Rac1 acts as an upstream molecule to induce actin polymerization, leading to Src activation in macrophages during inflammatory responses.

## Discussion

Many molecules are involved in the signaling pathways of inflammatory responses in macrophages (43–46). However, their roles and the underlying molecular mechanisms are still largely unidentified. The functional relationship between the key molecules, such as Syk, MyD88, and Src, in inflammatory responses are also poorly understood. The present study explored the roles and the functional cooperation of Syk and MyD88 as well as the underlying molecular mechanisms of inflammatory responses in macrophages.

We first attempted to identify which molecule is a prime intracellular activator of the NF-κB signaling pathway in macrophages during an inflammatory response. Because Syk, Src, and Akt reportedly play a pivotal role in the activation of inflammatory responses in macrophages (6, 8, 10), these molecules were examined in LPS-stimulated RAW264.7 cells to determine which one is a prime activator of the NF-κB signaling pathway. Of these molecules, Syk was a key activator of the NF-κB activity (**Figure 1A**), inducing the activation of signaling molecules and transcription factors p65 and p50 in the NF-κB signaling pathway in LPS-stimulated RAW264.7 cells (**Figures 1B–D**). Syk was activated immediately at 1 min, while p85 and IκBα were activated at 3 min and 5 min, respectively (**Figures 1B–C**), whereas nuclear translocation of two NF-κB subunits, p65 and p50, was markedly induced at 15 min in response to LPS in RAW264.7 cells (**Figure 1D**), which indicates that Syk is an upstream activator of p85 and IκBα, leading to the activation of the NF-κB signaling pathway in macrophages during inflammatory responses. We further confirmed the role of Syk in macrophage-mediated inflammatory responses using Syk-deficient RAW264.7 cells. A Syk-deficient condition in RAW264.7 cells was generated in two ways: Syk knock-out (Syk^−/−^) by CRISPR Cas9 technology and Syk inhibition by selective Syk inhibitors, Pic and Bay. Regardless of the Syk-inhibition mechanism, Syk inhibition significantly suppressed the NF-κB signaling pathway in LPS-stimulated RAW264.7 cells (**Figures 1E–I**). These results suggest that Syk activates the NF-κB signaling pathway by triggering downstream effector molecules p85 and IκBα at early time points (3 and 5 min, respectively), resulting in the activation of NF-κB transcription factors at 15 min in macrophages during inflammatory responses.

MyD88 and TRIF are intracellular adaptor molecules that interact with the cytoplasmic domain of TLR4 and play a crucial role in the induction of TLR4-induced signal transduction cascades in macrophages during inflammatory responses (8, 41). We therefore investigated the functional relationship between MyD88/TRIF and Syk in macrophages during inflammatory responses. Among MyD88 and TRIF, only MyD88 was involved in IκBα phosphorylation at 5 min (**Figure 2A**). IκBα was activated at an early point (5 min) in the response to Pam3CSK4, a molecular ligand of TLR2 that interacts only with MyD88 and LPS (a molecular ligand of TLR4 that interacts with both MyD88 and TRIF), while IκBα activation was markedly delayed (by 60 min) in response to poly(I:C), which is a molecular ligand of TLR3 that interacts only with TRIF (**Figure 2B**). Given the evidence that Syk activates IκBα early under LPS stimulation in RAW264.7 cells (**Figures 1B–C**), these results suggest functional cooperation between Syk and MyD88 rather than TRIF in macrophages during inflammatory responses. Because MyD88 is an adaptor molecule of TLR4, MyD88 can be expected to be an upstream molecule that activates Syk under inflammatory conditions in macrophages. We confirmed that MyD88 induced Syk activation and Syk-mediated NF-κB activity (**Figures 2C–E**). As expected, Syk KD was an essential domain for Syk-mediated NF-κB activation (**Figure 2E**). The NF-κB signaling pathway plays critical roles in the activation of inflammatory responses, such as ROS generation, upregulation of expression of inflammatory enzymes and pro-inflammatory cytokines, and induction of phagocytic activity in macrophages (11–16). In our previous study, Syk induced ROS generation, expression of ROS-generating enzymes, and phagocytic activity in macrophages during inflammatory responses (unpublished data). Given that MyD88 activated Syk and Syk-induced NF-κB activity in macrophages (**Figures 2C–E**), we generated MyD88^−/−^ RAW264.7 cells and examined whether MyD88 also induces ROS generation, expression of ROS-generating enzymes, and phagocytic activity in these cells during inflammatory responses. As expected, MyD88 induced ROS generation, expression of ROS-generating enzymes, and phagocytic activity in macrophages during inflammatory responses, and inhibition of MyD88 abolished these responses in MyD88^−/−^ RAW264.7 cells (**Figures 2F–H**), strongly suggesting that MyD88 is an upstream activator of Syk that induces Syk-mediated ROS generation, expression of ROS-generating enzymes, and phagocytic activity by activating the NF-κB signaling pathway in macrophages during inflammatory responses.

As discussed earlier, MyD88 is an upstream activator of Syk in macrophages during inflammatory responses. However, the molecular mechanism by which they cooperate in these inflammatory processes remains unclear. We therefore investigated the functional relationship between Syk and MyD88, and the molecular interaction between Syk and MyD88 was examined first. Syk interacted with MyD88 in RAW264.7 cells and peritoneal macrophages isolated from mice (**Figure 3A**). To examine which domains of Syk and MyD88 are essential for molecular interactions, domain-deletion mutants of Syk (**Figure 3B**) and MyD88 (**Figure 3C**) were generated, and their interaction was examined further. MyD88 interacted with Syk(ΔKD) but not with Syk(ΔSH2-N) and Syk(ΔSH2-C) (**Figure 3B**), indicating that the two SH2 domains of Syk are critical while the kinase domain of Syk is dispensable with respect to interactions with MyD88. In addition, Syk interacted only with WT MyD88, and none of the MyD88 mutants interacted with Syk (**Figure 3C**). This can be explained by two possibilities: the first is that all three domains of MyD88 are essential for the interaction with Syk, and the second is that deleting any one of these three domains could induce a significant conformational change, resulting in failure to interact with Syk. To clarify these possibilities and examine other possibilities, more studies are warranted. The immunoreceptor tyrosine-based activation motif (ITAM), which has the consensus sequence of YxxI/Lx_6-12_YxxI/L, is a conserved phosphorylation module found in a large number of adaptors and receptors that engages in a variety of biological processes and functions (47). Receptor ligation induces the tyrosine phosphorylation of the ITAM by intracellular kinase families, which in turn leads to the recruitment, interaction, and activation of Syk (48). After hemi-ITAM, a single copy of ITAM, was found in MyD88 (YLEI; 58 – 61), we turned our attention to the interaction of Syk with the MyD88 ITAM deletion mutant, MyD88(ΔITAM) and the point mutant of tyrosine in the ITAM to phenylalanine, MyD88(Y58F). Syk did not interact with both MyD88(ΔITAM) and MyD88(Y58F) (**Figure 3D**), indicating that hemi-ITAM and the tyrosine 58 residue in the hemi-ITAM of MyD88 is essential for interactions with Syk. In addition, the activation of Syk and the NF-κB-luciferase activity induced by MyD88 were markedly suppressed by MyD88(ΔITAM) and MyD88(Y58F) (**Figures 3E–F**). These results suggest that interaction of Syk with its upstream molecule, MyD88, is crucial for the activation of Syk and the subsequent NF-κB signaling pathway in macrophages during inflammatory responses, and that the hemi-ITAM and tyrosine 58 residues in the hemi-ITAM of MyD88 play essential roles in Syk-MyD88 interaction.

Receptor-ligand interactions induce the phosphorylation of the ITAM in receptors and adaptors to initiate signal transduction cascades in cells (48), which raised the question of whether MyD88 is phosphorylated at the tyrosine 58 residue in the hemi-ITAM for its activation during the macrophage-mediated inflammatory responses. MyD88 was immediately phosphorylated by LPS at 1 min (**Figure 4A**) in LPS-stimulated RAW264.7 cells, and the tyrosine 58 residue was identified as the main amino acid to be phosphorylated (**Figure 4B**). Several intracellular molecules, such as Src family kinases, TLR4, and its adaptors, have been identified as upstream molecules activated immediately by PAMPs and DAMPs to induce the inflammatory responses in macrophages (6–9). Which molecule is responsible for the phosphorylation of MyD88 at the tyrosine 58 residue was therefore investigated. To identify the upstream molecules that phosphorylate MyD88, RAW264.7 cells were co-transfected with MyD88 and either TLR4, Mal, or the Src family kinases, Src and Fyn. TLR4 and Mal induced MyD88 phosphorylation (**Figure 4C**), which is an inevitable result as MyD88 activation is induced by TLR4 and its adaptors (8). Among the Src family kinases, Src (but not Fyn) induced MyD88 phosphorylation (**Figure 4C**), indicating that they have distinct roles despite belonging to the same family, and that not all Src family kinases induce MyD88 phosphorylation and the subsequent MyD88-activated signal transduction cascades in macrophages during inflammatory responses. Therefore, Src-induced MyD88 phosphorylation was further investigated. Src was phosphorylated by LPS rapidly at 15 to 30 s, lasting up to 120 s (**Figure 4D**), indicating that Src is activated by phosphorylation faster than MyD88 phosphorylated by LPS at approximately 1 min (**Figure 4A**) and that Src might be an upstream activator of MyD88 in macrophages. In accordance with the previous result (**Figure 4C**), Src induced MyD88 phosphorylation, while MyD88 phosphorylation induced by Src was abolished by the Src kinase domain-deletion mutant, Src(ΔKD) in RAW264.7 cells (**Figure 4E**). Endogenous Src-induced MyD88 phosphorylation was inhibited by loss of function of Src by the treatment of PP2 (**Figure 4F**). Src-induced MyD88 phosphorylation was also abolished by MyD88(Y58F) (**Figure 4G**). These results suggest that Src acts as an upstream activator of MyD88 by phosphorylating MyD88 at the tyrosine 58 residue in the hemi-ITAM during macrophage-mediated inflammatory responses.

**Figure 6:**
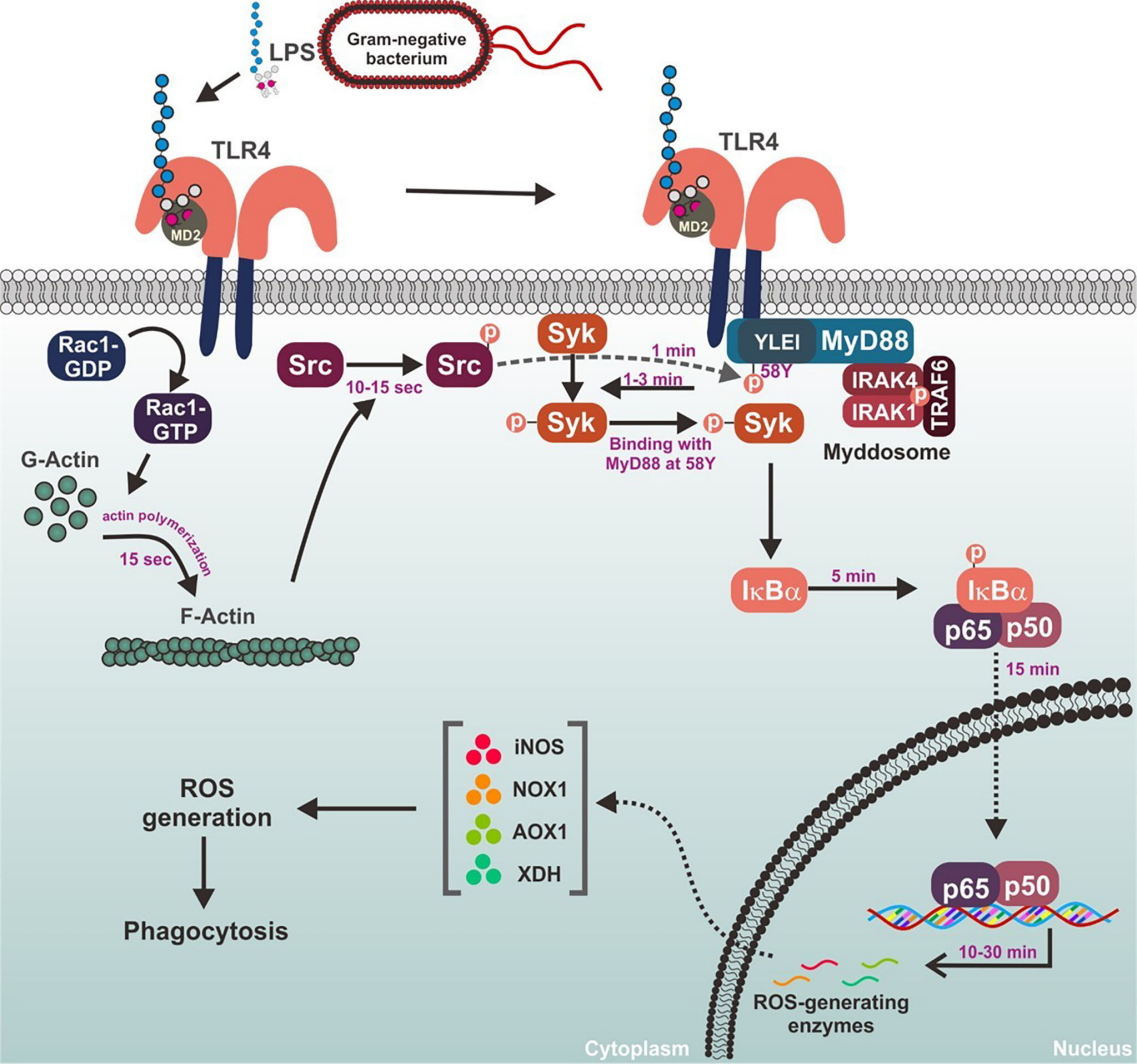
Schematic summary demonstrating the role of MyD88-Syk axis in the macrophage-mediated inflammatory responses.

The mechanism by which Src is activated is well established: dephosphorylation at the tyrosine 527 residue by various tyrosine phosphatases and the subsequent autophosphorylation at the tyrosine 416 residue in the kinase domain. However, which molecules are involved in and responsible for Src phosphorylation remains unclear, prompting an investigation of how Src is activated in macrophages during inflammatory responses. A previous study demonstrated that polymerization of globular actin to F-actin is a critical process in the inflammatory response (49–51). It also reported that disruption of the F-actin cytoskeleton structure induces Src deactivation by suppressing the formation of a signaling complex consisting of Src and p85/PI3K (52, 53), suggesting that inflammatory signals induce the formation of F-actin, and that F-actin subsequently activates Src, leading to the induction of the inflammatory signal transduction cascades in macrophages. To examine this, RAW264.7 cells were stimulated by LPS, and F-actin formation was evaluated. LPS immediately induced the formation of F-actin at 15 s, and the formation of F-actin was decreased after 15 s in RAW264.7 cells (**Figure 5A**). CytoB, a selective inhibitor of actin-filament formation, markedly inhibited Src phosphorylation induced by LPS in RAW264.7 cells (**Figure 5B**), leading to the subsequent inhibition of Syk phosphorylation in LPS-stimulated RAW264.7 cells (**Figure 6C**). These results suggest that actin polymerization is induced immediately in macrophages during the inflammatory responses, resulting in the activation of Src to transduce downstream inflammatory signals. Although F-actin activates Src by phosphorylation, F-actin is not a kinase; therefore, F-actin could indirectly activate Src by phosphorylation by inhibiting the formation of an inflammatory signaling complex composed of Src and p85/PI3K, as previously discussed (52, 53), or by activating other Src-phosphorylating kinases. Further research on this subject is needed. Rac1, a GTPase belonging to a Rho protein family, is implicated in various biological processes, such as fibrosis, apoptosis, gene regulation, and tumorigenesis (54, 55). Rac1 also reportedly plays a role as an upstream regulator in inducing inflammatory responses by increasing ROS generation and activating the NF-κB signaling pathway in response to a number of stimulations (54, 56). Therefore, the role of Rac1 in Src activation was further investigated. Similar to F-actin, Rac1 was activated immediately by LPS from 15 s to 180 s (**Figure 5D**), and inhibition of Rac1 suppressed Src phosphorylation induced by LPS in RAW264.7 cells (**Figure 5E**). In accordance with the previous observation that Rho GTPases, including Rac1, induce actin polymerization (57, 58), inhibition of Rac1 activation by NSC23766 suppressed the formation of F actin (**Figure 5F**) and the phosphorylation of both Src and Syk in the LPS-stimulated RAW264.7 cells (**Figures 5G–H**). These results suggest that Rac1 induces the polymerization of actin, leading to the subsequent activation of Src in macrophages during inflammatory responses.

Although this study demonstrates that MyD88-Syk axis is a key determinant in the activation of inflammatory responses through molecular interactions and functional cooperation and regulated by upstream Src kinase activated by Rac1-induced F-actin formation in macrophages, some related studies with different findings were also reported. Integrin CD11b-activated Syk interacted with MyD88 and TRIF, leading to the degradation of these adaptor molecules and the suppression of TLR-triggered inflammatory responses in macrophages (59). Syk was also reported to play a different role in TLR4-mediated inflammatory responses as a common regulator of various TLR responses. Syk oppositely regulated the TLR4-mediated inflammatory responses by inhibiting MyD88-dependent, but by enhancing TRIF-dependent inflammatory responses in macrophages (60). Moreover, Syk was reported to play a divergent role by exerting both TLR4-mediated pro- and anti-inflammatory in dendritic cells (61).

In conclusion, Syk is a prime activator of inflammatory responses that stimulates the NF-kB signaling pathway in macrophages. Under inflammatory conditions, Syk is immediately activated by molecular interactions with its upstream adaptor, MyD88, and the tyrosine 58 residue in hemi-ITAM of MyD88 was identified as an essential site for their interaction in macrophages. MyD88-Syk axis is activated by the upstream kinase Src through the phosphorylation of MyD88 at the tyrosine 58 residue, and Src is activated by Rho-GTPase Rac1-induced F-actin formation in macrophages. The role of the MyD88-Syk axis during the inflammatory responses in macrophages is summarized in **Figure 6**. These observations contribute to our understanding of the meaning and significance of the early activation of the MyD88-Syk axis and provide a mechanistic basis for these events in macrophage-mediated inflammatory responses. Because the MyD88-Syk axis activates rapidly in macrophages during inflammatory responses, selective targeting of one or both of these molecules may be a promising strategy to prevent and treat inflammatory and autoimmune diseases.

## Data Availability Statement

The original contributions presented in the study are included in the article/supplementary material. Further inquiries can be directed to the corresponding authors.

## Ethics Statement

Preparation of primary macrophages was performed in agreement with the guidelines of the Institutional Animal Care and Use Committee Sungkyunkwan University (Suwon, Korea; approval ID: SKKUIACUC2020-06-30-1).

## Author Contributions

Y-SY, HK, JK, and JC conceived and designed the experiments. Y-SY, HK, JK, WY, EK, DJ, JP, and NA, performed the experiments. Y-SY, HK, JK, SK, NP, and JC analyzed the data. SK and NP contributed reagents/materials/analysis tools. Y-SY, HK, JK, and JC wrote, reviewed, and edited the manuscript. All authors have read and agreed to the published version of the manuscript.

## Funding

This research was funded by the National Research Foundation of Korea (NRF), the Ministry of Education (2017R1A6A1A03015642), Korea.

## Conflict of Interest

The authors declare that the research was conducted in the absence of any commercial or financial relationships that could be construed as a potential conflict of interest.

## Publisher’s Note

All claims expressed in this article are solely those of the authors and do not necessarily represent those of their affiliated organizations, or those of the publisher, the editors and the reviewers. Any product that may be evaluated in this article, or claim that may be made by its manufacturer, is not guaranteed or endorsed by the publisher.
